# The Hepatitis E virus intraviral interactome

**DOI:** 10.1038/srep13872

**Published:** 2015-10-14

**Authors:** Andreas Osterman, Thorsten Stellberger, Anna Gebhardt, Marisa Kurz, Caroline C. Friedel, Peter Uetz, Hans Nitschko, Armin Baiker, Maria G. Vizoso-Pinto

**Affiliations:** 1 Max von Pettenkofer-Institute, Department of Virology, Ludwig-Maximilians-University, Munich, Germany; 2 Institute of Toxicology and Genetics, Karlsruhe Institute of Technology, Karlsruhe, Germany; 3 Bavarian Health and Food Safety Authority, Oberschleissheim, Germany; 4 Institute for Informatics, Ludwig-Maximilians-University, Munich, Germany; 5 Center for the Study of Biological Complexity, Virginia Commonwealth University, Richmond, VA, USA; 6 Instituto Superior de Investigaciones Biológicas (INSIBIO), CONICET-UNT, and Instituto de Fisiología, Facultad de Medicina, UNT. San Miguel de Tucumán, Argentina

## Abstract

Hepatitis E virus (HEV) is an emerging virus causing epidemic acute hepatitis in developing countries as well as sporadic cases in industrialized countries. The life cycle of HEV is still poorly understood and the lack of efficient cell culture systems and animal models are the principal limitations for a detailed study of the viral replication cycle. Here we exhaustively examine all possible intraviral protein-protein interactions (PPIs) of HEV by systematic Yeast two-hybrid (Y2H) and LuMPIS screens, providing a basis for studying the function of these proteins in the viral replication cycle. Key PPIs correlate with the already published HEV 3D structure. Furthermore, we report 20 novel PPIs including the homodimerization of the RNA dependent RNA polymerase (RdRp), the self-interaction of the papain like protease, and ORF3 interactions with the papain-like protease and putative replicase components: RdRp, methylase and helicase. Furthermore, we determined the dissociation constant (K_d_) of ORF3 interactions with the viral helicase, papain-like protease and methylase, which suggest a regulatory function for ORF3 in orchestrating the formation of the replicase complex. These interactions may represent new targets for antiviral drugs.

Hepatitis E virus (HEV) is the causative pathogen of Hepatitis E disease, a major form of acute viral hepatitis worldwide. Four different genotypes are known in various geographical regions^1^: genotype 1 (Asia), genotype 2 (Africa and Mexico), genotype 3 (Europe and North America) and genotype 4 (Asia). The global burden of genotype 1 and 2 infections is substantial and the number of infections is estimated at 20 million per year, leading to 70,000 deaths and 3,000 stillbirths^2^. The Hepatitis E virus is a single strand plus RNA virus belonging to the family *Hepeviridae*. Approximately 7,200 nucleotides encode for three open reading frames (Fig. 1). ORF1 is a nonstructural, functional poly-protein with several defined functions^3^,^4^. The first functional domain encodes a methyltransferase (Met), which is required for capping the 5′ end of the HEV RNA. Next, ORF1 contains a so-called Y domain which shows significant similarities to sequences of Rubella virus and Beet necrotic yellow vein virus but whose function in the viral context remains unknown. Further downstream ORF1 contains a papain-like cysteine protease (Plp) domain. The functionality of the protease domain was often questioned but recently the processing of the ORF1 poly-protein and ORF2 capsid protein by the Plp protease has been confirmed^5^. Next, a hypervariable (V) proline-rich region is located between the putative protease domain and the subsequent so-called X domain (X). For the proline-rich domain an important role in the fine tuning of viral replication through protein-protein interactions (PPIs) has been postulated^6^. The above mentioned X domain is also known as macro-domain or ADP-Ribose binding domain and has been exclusively associated with viral protease domains in other viruses. Towards the 3′ end, ORF1 encodes for the well characterized helicase domain (Hel) with NTPase and RNA unwinding activities, followed by the largest ORF1 domain, the RNA dependent RNA polymerase (RdRp). Second, ORF2 encodes for the viral capsid protein. X-ray crystal structure analysis lead to a sub-division of ORF2 in three subdomains: The S domain builds the capsid shell on which P1 and P2 form protruding spikes likely to be responsible for polysaccharide binding and antigenicity determination^7^. Dimerization and formation of virus-like particles (VLP) has been described for N-truncated forms of recombinantly expressed ORF2 protein where amino acids 126-601 constitute the essential elements required for self-assembly^8^. Third, ORF3 encodes a protein whose proposed function has been related to viral infection and replication based on some interactions with certain cellular proteins like microtubule subunits, MAPK phosphatase or Src homology 3 domain^4^,^9^. However, the life cycle of HEV is still poorly understood and the lack of efficient cell culture systems and animal models are the principal limitations for a detailed study of the viral replication cycle.

A modern approach to better understand a viral infection at the cell level is the use of systems biology tools to describe virus-host and intraviral PPIs, which are the key to most biological processes^10^. At present only a few intraviral interactomes of human pathogenic viruses are available in the literature: Epstein Barr virus (EBV)^11^,^12^, influenza virus^13^, hepatitis C virus (HCV)^14^,^15^,^16^, herpes simplex virus 1 (HSV-1)^11^,^17^,^18^, Kaposi’s sarcoma-associated herpesvirus (KSHV)^19^,^20^, SARS coronavirus^21^,^22^, vaccinia virus^23^, varicella zoster virus (VZV)^19^,^24^, and Chandipura virus^25^. So far only a few individual PPIs of HEV proteins have been reported including the self-association of ORF2p^7^,^26^,^27^, the self-interaction of ORF3p^28^ and an interaction between the ORF2p and ORF3p^29^ (Fig. 2a). In order to provide the first genome-wide map of intraviral HEV PPIs, we performed a high-throughput Y2H (yeast two-hybrid) screening of HEV ORFs and individual protein domains. To further improve the quality of the Y2H primary screening, systematic permutations of fusion proteins were used^24^. In a second step, interactions were verified using a modified luminescence-based mammalian interactome mapping pull-down assay (LuMPIS)^30^. LuMPIS, which is based on mammalian cells, likely better reflects genuine PPIs of proteins which undergo post-translational modifications rather than PPIs detected in yeast. Furthermore, we investigated the binding kinetics and characterized interactions of ORF3p using microscale thermophoresis (MST)^31^,^32^,^33^,^34^

## Results

### Identification of HEV PPIs by Yeast two-hybrid

We used an improved Y2H screen for mapping the HEV PPIs and performed it in two separate steps. First, 10 ORFs and ORF domains (ORF2 domains excluded) were tested in four different combinations of bait and prey vectors. This resulted in four different interaction subsets (Table 1) similar to those from other screens that had been performed with this improved Y2H system^24^,^35^,^36^. The pGBKT7g/pGADT7g vector pair, for example, yielded 31 redundant interactions while the combination of pGBKCg and pGADCg resulted in only 10 interactions. The quality of the Y2H interaction data was assessed both by counting redundancies among screens and retesting the Y2H interactions in an independent system, LuMPIS^30^. Taken together, 24 of 35 investigated single interactions (69%) were rated as “high quality” interactions, as they were detected in both Y2H and LuMPIS, which are based on different principles for the detection of PPIs (Table 2). The remaining 11 PPIs (31%) were classified as “medium quality” interactions with redundancies in either the Y2H or the LuMPIS data. Interactions defined as “basic quality”, i.e. found in either Y2H or LuMPIS but without redundancies, were not obtained in this screen (Table 2). All 10 protein coding ORFs and ORF1 domains interacted at least with one of the domains or full-length proteins tested. The protein interaction network of HEV is shown in Fig. 2b. The network is made up of 25 unique interactions with respect to the ten tested constructs, excluding the ORF2 self-interaction which will be described in the next section.

Besides the ORF2 self-interaction, we also detected for the first time the self-interactions of RdRp and Plp.

### ORF2 self-interaction

**Figure 1:**
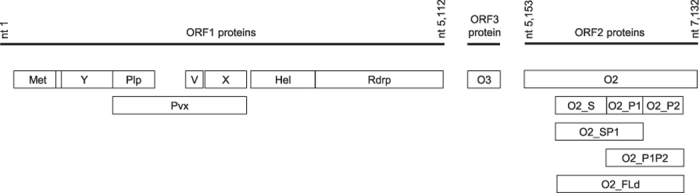
Schematic overview of all Hepatitis E virus ORFs and ORF subdomains. Bars indicate defined domains: Met: Methyltransferase; Y: Y-domain; Plp: Papain like protease; V: variable region (poly-proline-hinge); X: X-domain (macro-domain); Pvx: Protein consisting of the domains Plp, V and X; Hel: Helicase; Rdrp: RNA dependent RNA polymerase; O3: ORF3 protein; O2: ORF2 protein (capsid); O2_FLd: Full length delta; O2_S: shell domain; O2_P1: protruding domain 1; O2_P2: protruding domain 2; Nucleotide (nt) numbers indicating position in the sequence (adapted from Osterman *et al.*^54^).

Tyagi *et al.*^37^ showed the self-interaction of full-length ORF2 using a classical Y2H, which we could not reproduce in our permutated Y2H screening. Therefore, we sub-cloned three ORF2 domains, i.e.: S, P1 and P2 and combinations thereof (Fig. 1, Table 3) and performed a second permutated Y2H screening for protein interactions only for ORF2 domains. This showed that the truncated ORF2 proteins interacted with itself as well as with several ORF2 domains (Table 3). In contrast, in the mammalian PPI screen (LuMPIS) only the expression vector encoding the full length ORF2 protein revealed the self-interaction of the capsid protein. Our results further confirm this direct self-interaction of P2 (Table 2), and also validate the hypothesis that the P1 domain, which has been attributed a linker function, does not interact directly with P2. Therefore, the P1P2-P1P2 interaction (Table 3) is mediated by the self-interactions of P1, and P2, respectively. We first report the direct interactions S-P1, and P2-SP1, but a direct interaction between the ORF2 S-domains could not be confirmed in this study.

### Identification of HEV PPIs by LuMPIS

In total, 29 different interactions of HEV full length ORFs and ORF domains were tested in a secondary screening using LuMPIS (Fig. 3). Interactions were defined as positive when the Dunnett’s multiple comparison test showed significant differences between the LIR (luminescence intensity ratio) of sample and control interactions (p < 0.01). 25 out of 29 evaluated LuMPIS interactions were already tested as positive in the primary Y2H screening described above. In LuMPIS, PPIs were tested in two combinations using N-MBP and N-eGFPLuc tagged proteins. Thus, LuMPIS was mainly used here as a confirmatory assay. Additionally, four interactions previously described or hypothesized in the literature, i.e.: ORF2-ORF2^26^,^27^ , ORF2-ORF3^29^, ORF2-Plp and ORF2-Pvx were retested and compared to our Y2H results as these reported interactions could not be detected in the Y2H screening. These additional experiments studying full length ORF2 interactions lead us to the following results: an O2-O2 self-interaction and an O2-O3 interaction were only detected by LuMPIS, but no interaction was found between O2 and the protease domain, neither using the Y2H system nor the LuMPIS platform. In total, LuMPIS detected the self-interactions of five proteins (Plp, X, Pvx, O3, O2). Furthermore, all proteins had at least one interaction partner and O3 showed the highest number of interactions (eight) of all tested HEV proteins. The interaction of ORF3 with the protease domain was then further characterized using microscale thermophoresis. To sum up, 80% of the PPIs detected by the primary Y2H screen (20 of 25) were confirmed by LuMPIS. Y2H detected 25 out of 55 possible interactions (45%) between 10 HEV ORFs and ORF1 domains in the primary screening, whereas LuMPIS detected 20 of 55 possible interactions (36%) in a secondary confirmation test (Fig. 3). Next, interactions of the protease domain were explored in two different settings. On the one hand the protease domain was evaluated alone (Plp), and on the other hand in association with the V- and X-domains. Interestingly, the combination of eight interactions detected by the independent domains Plp, V, and X together can explain only partially the six interactions detected by the protein complex Pvx. However, all the interaction partners of Plp also interacted with Pvx. Here we show that Plp interacts with Plp itself, with Pvx and also with O3.

### Network properties

**Figure 2:**
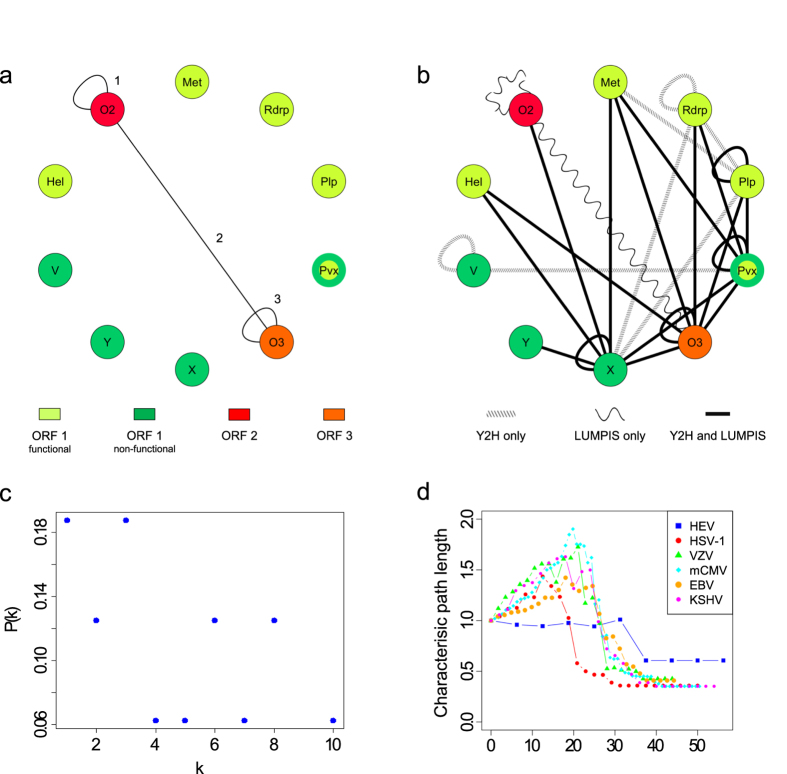
Protein-protein interactions in HEV and network properties. (**a**) Intraviral protein-protein interaction network of HEV according to literature. Green: ORF1 proteins, red: ORF2 proteins, orange: ORF3 proteins. Interactions: **1**) as reported by Li *et al.*^26^, Yamashita *et al.*^27^, Guu *et al.*^7^, **2)** as reported by Tyagi *et al.*^29^, and **3)** as reported by Tyagi *et al.*^28^ (**b**) Intraviral protein-protein interaction network of HEV detected by Y2H and/or LuMPIS. Proteins are colored as in A. The network was generated using Cytoscape (www.cytoscape.org). (**c**) Degree distribution of the HEV interactions obtained in the Y2H screening. The y-axis shows the frequency of proteins having the number of interactions shown on the x-axis. (**d**) Simulation of deliberate attack on HEV in comparison to five herpesviral Y2H networks by removing their most highly connected nodes in decreasing order. After each node is removed, the new network characteristic path length (average distance between any two proteins) of the remaining network is computed and plotted as a multiple or fraction of the original parameters. As the increase in path length is somewhat smaller than for the herpesviral networks, this may suggest a higher attack tolerance but may also be an artefact due to the small size of the networks.

Standard network analysis was performed using the PPI results obtained in both Y2H and LuMPIS. The analysis included the calculation of network properties such as average degree, characteristic path length and clustering coefficients. Results were compared both to intraviral interaction networks for SARS^21^ and five herpesviruses (HSV-1, VZV, murine CMV, EBV and KSHV)^11^,^19^,^38^ as well as two types of random networks of the same size (Table 4). On average, screened constructs showed ∼4.4 interactions. The characteristic path length was reduced compared to those found in other viruses. Finally, the clustering coefficient was increased (0.71–0.77) compared to the other viral networks (0.15 to 0.41) and, in particular, slightly increased compared to random networks with the same degree distribution. For the Y2H interaction network, we also calculated degree distributions and attack tolerance and compared them to published results of five herpesviruses (Fig. 2d). Due to the small size of the network, a proper fit of the degree distribution could not be performed. Nevertheless, a weak trend was observed towards a concentration of interactions within a few constructs. Attack tolerance appeared to be somewhat higher than for the herpesviral Y2H networks.

### Biophysical analysis of ORF3 interactions by microscale thermophoresis

The binding affinity of ORF3 with its interaction partners (Hel, Met, Plp, Pvx and X) were determined using a novel approach called microscale thermophoresis (MST)^31^,^33^,^34^,^39^,^40^. These proteins were expressed in *E. coli* as N-MBP-tagged proteins in order to be purified under native conditions using amylose beads. As a result, we could establish the following binding hierarchies of ORF3 and its different partners: Plp (1.5 nM) > Pvx (1.6 nM) > X (5.6 nM) > Met (33.7 nM) > Hel (87.9 nM) (Fig. 4a–e).

The ORF3 vs. Plp interaction was further characterized by studying the effect of temperature and pH on the binding strength. Figure 5a shows a clear temperature inverse dependency for the ORF3 vs. Plp binding: 30 °C (0.3 nM) > 35  °C (2.8 nM) > 37 °C (16 nM) > 41 °C (19 nM). Analogously, the strongest binding was detected at a pH of 7 (EC50: 0.3 nM) (Fig. 5b).

## Discussion

Y2H belongs to the most powerful tools for high-throughput screening of PPIs, though it is prone to relatively high rates of false negative and false positive PPIs. Although the false-negative rate of Y2H screens can be measured by sets of so-called “gold-standard” interactions^35^, it is much more difficult to estimate the rate of false positives. More importantly, false positive rates are largely dependent on the proteins used and the difficulty to determine the biological significance of PPIs^41^. In order to reduce the rate of false negative and false positive PPIs, we used an improved permutated assay^24^. Traditionally, vectors used for Y2H lead to synthesis of bait and prey proteins fused C-terminally to the Gal4 DNA-binding (DBD) and activation domains (AD). Recently, Stellberger *et al.*^24^ introduced two new Y2H vectors allowing the systematic testing of four different bait and prey combinations, here denoted as NN, NC, CC and CN with the two letters corresponding to N- or C-terminal fusion proteins of bait and prey, respectively. This improved Y2H system has a reduced rate of false negative PPIs, and the four vector combinations allow for better distinction of true (reproducible) and false (non-reproducible) positive PPIs.

Thirty-one PPIs were detected using the pGBKT7g/pGADT7g vector pair, whereas the pGBKCg/pGADCg vector pair allowed for the detection of twenty one of these interactions. Thus, the combination of these four different bait and prey vectors allowed us to examine redundant interactions with clear differences in the number of detected interactions. Therefore, we hypothesize that the differences observed may be a consequence of structural constraints resulting from the location of the DBD and AD tags^24^. In order to reduce the probability of negative results due to structural constraints, we also subcloned overlapping fragments of the predicted proteins. This again provides redundancy, which strengthens positive results and it may also help define domains mediating interactions. Due to the small number of proteins composing the HEV proteome, even though we included redundant test constructs, such as Plp, V, X and Pvx (Fig. 1), statistical analysis was still limited compared to data of bigger genomes. On the other hand, it allowed a detailed evaluation of the quality of interactions. Furthermore, as already pointed out by McCraith *et al.*^23^, the likelihood of a non-significant PPI occurring by chance is considerably lower in a smaller set of genes compared with the screening of large libraries with thousands of genes.

To determine sensitivity and specificity of the Y2H primary screen we used a completely independent system for secondary screening – LuMPIS^30^. Results obtained with LuMPIS confirmed two thirds of all the interactions detected in the Y2H screen. This underlines the quality of this data set. In addition, we describe for the first time the intraviral PPI network of Hepatitis E virus setting a reliable fundament for further research.

A couple of expected PPIs of HEV were detected and allowed further insight into virus replication. This includes the self-interaction of RdRp which is in line with data obtained for other viral replicases of (+) stranded RNA viruses such as Poliovirus^42^ and Chikungunya virus^43^. It has been postulated that this self-interaction is important for polymerase function^42^.

We also first describe the Plp self-interaction and its interaction with other nonstructural proteins such as the methylase, RdRp and ORF3. These PPIs suggest that Plp may have dual roles in virus life cycle, i.e.: apart from cleaving ORF1 polyprotein, it may also be involved together with ORF3 in assembly of replication complexes. This is in accordance to previous results on the protease activity of Plp reported by Karpe and Lole^44^ and with the hypothesis that ORF1 processing, occurs only in the context of HEV replication^44^,^45^. In line with our results, Purdy and Khudyakov^6^ found an intrinsically unstructured region in the ORF1 polyprotein which allows binding to multiple ligands, including proteins.

ORF2 self-interactions have been repeatedly described in the literature including structural analysis that suggests interactions of ORF2 subdomains. All positive-stranded RNA eukaryotic viruses described until now seem to have a capsid protein which is folded as β-barrels with jelly roll topology^46^,^47^. Typically, this capsid protein comprises a tightly closed shell domain (whose function is to protect the viral RNA) and a poorly conserved projection domain^48^,^49^. Based on the HEV ORF2 and VLP crystal structures, Guu *et al.*^7^ proposed that ORF2p is composed of three domains: S, P1 and P2. Each one constitutes a different structural element with S constituting the continuous capsid, P1 the 3-fold protrusions, and P2 the 2-fold-spikes. Furthermore, the N-terminal region of ORF2 may represent the shell domain, whereas the C-terminal region constitutes the projection domain. According to Guu *et al.*^7^, when full-length ORF2 is expressed in insect cells, the first 111 amino acids are proteolytically removed before dimerization and assembly of virus-like particles (VLPs). In addition, other authors showed that truncated forms of the ORF2 protein oligomerize^8^,^26^,^50^. This is in line with our results, as we confirmed the postulated dimerization of ORF2 full-length protein using LuMPIS.

On the other hand, the classical Y2H detected multiple interactions among ORF2 domains S, P1 and P2 (Table 3). Guu *et al.*^7^ showed in the HEV VLP crystal structure that P2 forms a dimer, which constitutes the spike on the surface of the capsid and was confirmed in our study. Besides the P1P2-P1P2 interactions described above, we first report a direct interaction between P1 and S, which suggests that P1 may be responsible for stabilizing S during shell assembly as a linker domain. S as a bait construct did not interact in the Y2H-screening neither as an N- nor as a C-terminally tagged construct with any of the tested proteins. Therefore, a direct interaction between the ORF2 S-domains and the formation of a continuous shell as predicted from the 3-D structure^8^,^26^,^50^ could not be confirmed in this study. Additionally, we have to keep in mind that the false negative rate of our Y2H screening method is at least 20%^35^, which means that up to 80% of the true-positive HEV intraviral protein interactions may have been detected in this study.

**Figure 3:**
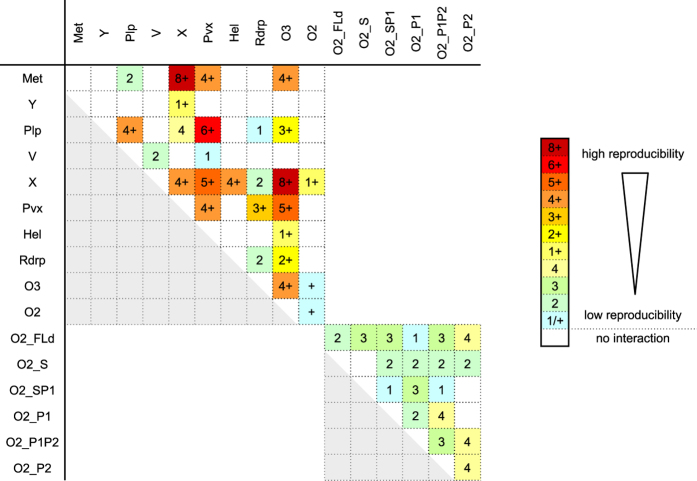
Overview of all intraviral protein-protein-interactions of HEV by primary Y2H and secondary LuMPIS screen. Data are presented as a heat map, ranging from dark red (high reproducibility) to light blue (low reproducibility). The numbers indicate how many Y2H vector combinations were positive (8 being the highest). (+) indicates a PPI positive result in the independent LuMPIS screen. White squares represent absent interactions.

**Figure 4:**
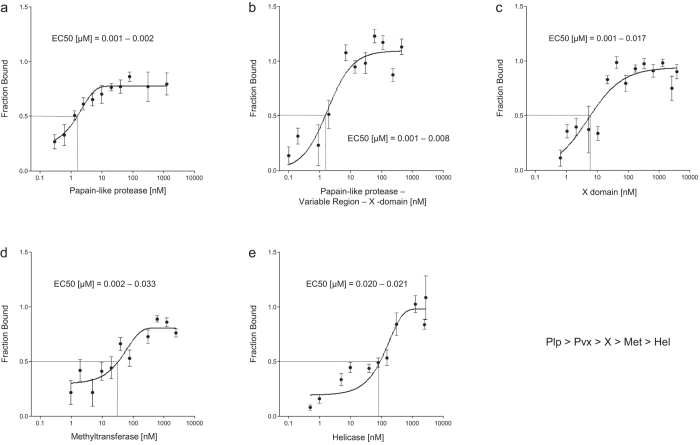
Binding affinities of ORF3. EC50 values indicate the concentration at which 50% of the interaction partners of ORF3-MBP-N were bound. Curves were calculated from two experiments with three measurements each. Binding curves of N-MBP-ORF3 interactions: (**a**) ORF3 vs. Plp; (**b**) ORF3 vs. Pvx; (**c**) ORF3 vs. X; (**d**) ORF3 vs. Met; (**e**) ORF3 vs. Hel. All measurements used N-terminal MBP fusions.

**Figure 5:**
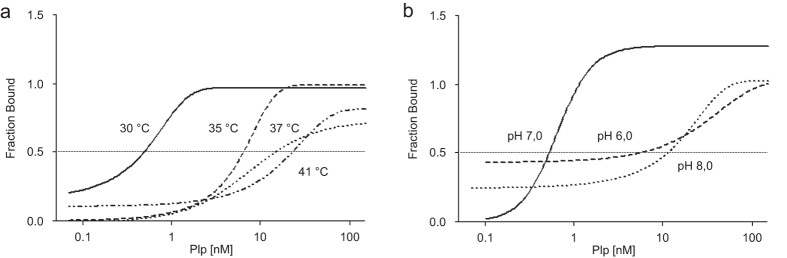
Temperature and pH dependency of ORF3-Plp interaction. Binding curves of ORF3-Plp interaction was measured by thermophoresis. (**a**) The EC50 is temperature-dependent: 30 °C: 0.3 nM, 35 °C: 2.8 nM, 37 °C: 16 nM, 41 °C: 19 nM. (**b**) The EC50 is pH- dependent: pH 6.0: 19 nM, 7.0: 0.3 nM, and 8.0: 6 nM.

As mentioned before, we used LuMPIS as a secondary screening platform to retest 29 positively detected interactions from the primary Y2H screen. Besides this confirmatory aspect, four HEV PPIs already described in the literature but not detected in our Y2H screening were examined (i.e.: ORF2-ORF2^8^,^50^, ORF2-ORF3^28^,^29^, ORF2-Plp and ORF2-Pvx (Fig. 3)). The ORF2-ORF3 interaction as well as the ORF2 self-interaction were confirmed by LuMPIS. However, an interaction between ORF2 and the protease, which are believed to interact during posttranslational cleavage to allow capsid formation of truncated ORF2 proteins, could not be detected. Nonetheless, we cannot rule out that the negative result may also be the consequence of the inherent proteolytic activity of the protease during the assay. When analyzing in more detail the PPIs detected by Y2H but not by LuMPIS, three of them (V-V and V-Pvx and Rdrp-Rdrp) were detected when the respective interaction partners were C-terminally tagged (Fig. 3). This suggests possible steric constraints due to the position of the respective tags. In the confirmatory LuMPIS screening only interactions between N-terminally tagged HEV proteins could be assessed as for this platform C-terminal vectors are not available yet. Whether the ORF1 encoded polyprotein itself contains multiple biochemical activities or whether it is processed to give distinct individual units has not been elucidated yet.

In this study, we showed that ORF3 binds to three viral proteins with distinct functions: the helicase, the methyltransferase and the proteinase as well as two proteins of unknown function; the variable region and the X-domain. The binding affinity of ORF3 with its interaction partners (Hel, Met, Plp, Pvx and X) were measured using a novel approach called microscale thermophoresis (MST). By monitoring the thermophoretic behavior of a fluorescence-labeled protein, it was possible to detect changes in the hydration shell caused by the binding of putative interaction partners. By titrating the interaction partners vs. a constant amount of the labeled protein (ORF3) we generated binding curves and determined the binding constant for ORF3 interactions which strongly suggest regulatory functions for this protein. The relevance of the ORF3-X domain interaction is difficult to assess as the biological relevance of the X domain is unknown.

Considering the EC50 values of the MST-experiments evaluating the temperature and pH dependency of the N-MBP-ORF3 and N-MBP-Plp interaction, the optimal conditions were 30 °C and a pH value of 7. The inverse temperature dependent relationship of K_d_ values does not necessarily reflect properties of viral proteins during replication cycle but only common thermodynamic findings of protein interactions. On the other hand the highest K_d_ at pH of 7 assumes an optimized design for effective interaction of ORF3 and Plp protein under neutral pH resembling cytoplasmatic conditions.

In general, standard network parameters obtained for HEV PPIs were consistent with previous reports on other virus networks. The average number of interactions was ∼4.4 per HEV protein, the average length of the shortest paths between any two nodes in the network was smaller than the diameter found for other viruses (SARS and Herpesviruses), and finally, the clustering coefficient was higher than in other reported viral networks and random networks with the same degree distribution. The attack tolerance was calculated by removing nodes (causing network failures) and calculating the characteristic path length after each removal. As a result, the HEV PPI network showed a higher robustness in comparison to SARS and herpesviruses networks. It has been hypothesized that attack tolerance explains the error tolerance of complex systems such as the cell^51^.

The HEV network seems not to be homogeneous as we identified a trend to a concentration of interactions within few HEV proteins. This may point at the Achilles’ heel of the viral network as these proteins play a vital role in maintaining connectivity. Therefore, identifying these highly connected nodes opens for us the possibility to establish targets for antiviral drug design.

In HCV, the virion is assembled in a multi-step process involving nonstructural proteins^52^. Analogously, we hypothesize that HEV also needs nonstructural proteins that orchestrate virion assembly. Thus, due to the multiple interactions detected for ORF3 with other HEV proteins, ORF3 may be involved in this process acting as a hub protein that connects proteins involved in assembly. This further supports the hypothesis that in small size viruses, viral replication is effective because the few proteins involved in the process are multifunctional. ORF3 does not only interact with several viral proteins but also 32 host proteins as reported by Geng *et al.*^53^ in a Y2H screen. The average degree of the HEV ORF3 vs. host interaction network is 8.3^53^ which doubles the average calculated for the intraviral network. Nevertheless, this is not surprising considering the small size of the HEV proteome compared to the liver proteome. Furthermore, we present evidence of a direct physical contact of ORF3 with at least three proteins: Hel, RdRP and Met, could be involved in the putative replicase complex and ORF3 may function as a hub protein coordinating assembly. Further functional studies are required to understand the mechanisms involved.

The HEV interactome sets up the basis for studying the function of these proteins in the viral replication cycle; the identified interactions may result in potential targets for new antiviral drugs.

## Methods

### Recombinatorial cloning of expression vectors of a complete HEV library

A complete genotype 1 HEV genome library served as template for recombinatorial cloning^54^. Ten HEV ORFs and ORF domains (Met, Y, Plp, V, X, Pvx, Hel, RdRp, O3, O2) were subcloned into Gateway® compatible vectors for Y2H^24^ , LuMPIS^30^ and bacterial expression, respectively. For Y2H studies ten ORFs were cloned into N-terminally tagged (pGBKT7g, pGADT7g) and ten C-terminally tagged (pGBKCg, pGADCg) vectors, respectively. For LuMPIS experiments 17 N-terminally tagged clones were made in pCR3.1NMBP and pCR3.1NeGFPluc^30^. Proteins Met, Plp, Pvx, X and Hel were expressed for thermophoresis experiments using pETGNMBP^55^. Domain sequences were defined as described recently^54^. In addition, the putative functional protease complex^3^ was subcloned (Pvx) consisting of the papain like protease, the proline rich domain (Y, serving as a link to the downstream X domain) and the X-domain, which is found exclusively in association with viral papain like proteases. Furthermore, for detailed Y2H study of ORF2 interactions six additional ORF2 domains analogous to the S, P1 and P2 domains as defined by Guu *et al.*^7^ were subcloned. Finally, two overlapping sequences (SP1, P1P2) and a N- and C-terminally truncated ORF2 sequence^27^ were cloned. For this additional Y2H experiments N- and C-terminally tagged expression vectors were used. All constructs are listed in Supplementary Table 1.

### Y2H experiments

Yeast two-hybrid assays were performed by an array-based strategy as previously described^24^. Briefly, bait constructs in the vector pGBKT7g were transformed into the reporter yeast strain Y187 (MATα, ura3-52, his3-200, ade2-101, trp1-901, leu2-3, 112, gal4Δ, met-, gal80Δ, URA3::GAL1_UAS_-GAL1_TATA_-lacZ) and prey constructs in pGADT7g into AH109 (MATa, trp1-901, leu2-3, 112, ura3-52, his3-200, gal4Δ, gal80Δ, LYS2::GAL1_UAS_-GAL1_TATA_-HIS3, GAL2_UAS_-GAL2_TATA_ADE2, URA3::MEL1_UAS_-MEL1_TATA_-lacZ). The resulting strains were grown on synthetic defined (SD) medium lacking either tryptophan (-trp) or leucine (-leu) and arrayed as quadruplicates in a 384 pin format using a Biomek 2000 workstation (Beckman-Coulter). By an all-against-all matrix approach each bait strain was mated against the whole prey array as well as against the empty prey vector strain. After one day at 30 °C the matings were replicated from rich medium (YEPD) to SD-leu-trp to select for diploids. After two days the diploid strains were transferred to selective SD-leu-trp-his readout plates to select for reporter gene activity. Every bait strain was assayed on the appropriate minimum inhibitory concentration of 3-amino-1,2,4-triazole (3-AT), a competitive inhibitor of pHIS3 in order to suppress unspecific background or the autoactivation properties of the individual bait proteins; i.e. if baits were autoactivating under 1 mM 3-AT, the concentration was raised stepwise (1, 3, 10, 25, and 50 mM). Yeast growth was evaluated after seven days at 30 °C.

### Luciferase-based detection MBP-pull down interaction screening (LuMPIS)

LuMPIS was performed as described before^30^. Briefly, semiconfluent HEK 293 T cells grown in 12-well plates were transfected with the respective prey and bait vectors using FuGENE HD™. After 48 h the cells were lysed in 500 μl lysis buffer (20 mM Tris-HCl, 200 mM NaCl, 1 mM EDTA, 0.05% Tween 20™, 5 μg/ml Leupeptin, 5 μg/ml DNAse I, 2.5 mg/ml BSA, pH 7.5) by sonication (5 pulses of 15 seconds) at 4 °C. Lysates were cleared by centrifugation at 13,000 g at 4 °C for 10 min and then diluted 1:10 in washing buffer (20 mM Tris-HCl, 200 mM NaCl, 1 mM EDTA). MBP tagged bait proteins were captured by using 100 μl of pre-equilibrated 50% slurry amylose beads (New England Biolabs, Germany). After washing the amylose beads four times with 200 μl washing buffer, the captured MBP tagged bait proteins were eluted with 150 μl 10 mM maltose using a vacuum Manifold (Millipore, Germany). The co-eluted eGFP-Luc tagged prey proteins were detected by measuring luciferase activity in 50 μl eluate after addition of 50 μl luciferase assay reagent (Promega, Germany) using an Optima FLUOstar Luminometer system (BMG LABTech, Germany). The Luminescence Intensity Ratio (LIR) was calculated as:


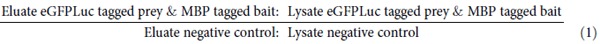


The negative control (i.e. eGFP-Luc tagged prey expression vector co-transfected with the empty MBP vector) was performed for each prey protein. Two independent experiments (in quadruplicate) were done. The data were statistically analysed by ANOVA (P < 0.05) followed by Dunnett’s post hoc test.

### Bioinformatic analysis

The following network properties were analyzed for the PPI networks: network size (=number of proteins and interactions, both including and excluding self-interactions), average degree (=number of interactions per proteins), characteristic path length (=average shortest path length between any pair of proteins), diameter (=maximum shortest path length), clustering coefficient and enrichment of clustering coefficient compared to the average of 1000 random graphs of the same size (ER) and 1000 random networks with the same degree distribution (ES). For the LuMPIS network, enrichment over ES could not be calculated due to its small size. Self-interactions were not included for the computation of average clustering coefficients, characteristic path length and network diameter as well as enrichment values. In addition, we calculated the degree distribution (P(k) = frequency of proteins with k interactions) and attack tolerance of the Y2H network. Attack tolerance was calculated by repeatedly removing the protein with the largest number of interactions from the network, recalculating characteristic path length and comparing it to the characteristic path length of the original network. A rapid increase of this characteristic path length indicates low attack tolerance.

### Microscale Thermophoresis

MST is a new biophysical technique to determine binding affinities in solution. Using an infrared laser a local microscale temperature gradient is produced and the thermophoretic movement of the molecules can be measured. The detailed background of this new method is described elsewhere^31^,^32^,^33^,^34^. In order to measure the thermophoresis, one of the interaction partners was labeled. The MBP-tagged and purified N-MBP-ORF3 protein was rebuffered in PBS (pH 8.3) added with 130 mM NaHCO_3_ and 50 mM NaCl using Zeba Spin Desalting Column (Thermo Fisher Scientific, USA) at 10 °C according to the manufacturer’s protocol. A 65 μl aliquot of the protein to be labeled was set to a concentration of 2–20 μM, mixed with an equal volume of a 3-fold higher concentrated aliquot of A_647_ (Life Technologies, USA) and incubated for 30 min at room temperature. The labeled protein was purified from free dye using again a Zeba Spin Desalting Column and PBS as destination buffer. Within the MST assay the fluorescence-labeled partner N-MBP-ORF3-A_647_ was kept in a constant end-concentration of 0.06 μM in PBS (pH 7) added with 0.02% Tween 20, 2% BSA and 0.1 mM maltose (N-MBP-ORF3-A_647_ vs. N-MBP-Hel, -Met, -Plp, and -X), whereas the putative binding partner was set at various concentrations in PBS by preparing 2-fold dilution series (the optimal concentration/fluorescence ratio was determined using the Monolith NT.015T (NanoTemper Technologies GmbH, Germany). Both, the labeled and the unlabeled partner were mixed in equal volumes and incubated at 30 °C for 30 min to reach equilibrium. The mixture (5–10 μl) was then measured in a Monolith NT.015T at 30 °C by using a red LED (LED-Power 100 V; Laser on time 25 s; Voltage 1 V) following manufacturer’s instructions. Due to the high sensitivity of MST for PPIs, the use of MBP as a negative interaction control revealed a very weak though existent interaction between N-MBP-ORF3 and MBP. Interestingly, there is one single published report that MBP can dimerize in 10 mM Tris-HCl, but except for this report, it is believed that MBP is a monomer^38^. As Hall *et al.* showed that MBP binds maltose in its active site resulting in a conformational change^56^, we tested if the presence of maltose at saturating concentrations (50 μM) was able to reduce MBP dimerization. Indeed, this was the case and we further proceeded to test the binding of all partners including the negative control (MBP alone) in the presence of maltose. For each interaction pair the data of two experiments with three measurements each were combined. The EC50 values were calculated based on the Hill equation using the Monolith NT.015T analysis software. In order to compare the different measurements, graphs were generated by GraphPad Prism 5.01. Therefore the signal of thermophoresis was normalized as “Fraction Bound”. The “Fraction Bound” is the quotient of the concentration of bound molecules to the concentration of constant provided fluorescence-labeled molecules calculated using the normalized fluorescence signal (Fnorm):


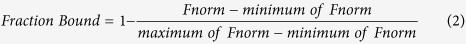


as described by Seidl *et al*^40^.

Unless indicated differently, experiments were performed at 30 °C and pH 7.

### Dataset

The protein interactions from this publication have been submitted to the IMEx (http://www.imexconsortium.org) consortium through IntAct^57^ and assigned the identifier IM-23809.

## Additional Information

**How to cite this article**: Osterman, A. *et al.* The Hepatitis E virus intraviral interactome. *Sci. Rep.* **5**, 13872; doi: 10.1038/srep13872 (2015).

## Supplementary Material

Supplementary Information

## Figures and Tables

**Table 1:** Vectors and Y2H PPI summary.

Bait/prey vectorcombination	Tag fusions	Intraviralinteractions^a^	ORF2 interdomaininteractions^a^
pGBKT7g/pGADT7g	NN (N-terminal fusions)	31	1
pGBKT7g/pGADCg	NC (N-terminal/C-terminal fusions)	23	16
pGBKCg/pGADT7g	CN (C-terminal/N-terminal fusions)	21	19
pGBKCg/pGADCg	CC (C-terminal fusions)	10	10

Tag fusions indicate the location of the DNA-binding (DBD) and transactivation domain (AD) of the resulting hybrid proteins in the corresponding vector combination.

^a^Redundant interaction sets. Included are interactions found with different vector combinations.

**Table 2:** Intraviral protein interactions from Y2H screening.

InteractionNo.	Bait	Prey	NN	NC	CN	CC	N°Vectorpairs	Redundant PPI inY2H (PPI No.)	Scoredon X mM3-AT	LuMPIS	PPIQuality
1	Met	X	+	+	+	+	4	r, 18	0	+	high
2	Met	O3	+	+	+	+	4		0	+	high
3	Met	Plp	+	−	+	−	2	4, 12	0	−	medium
4	Met	Pvx	+	−	+	−	2	r, 3, 12	0	+	high
5	Y	X	+	−	−	−	1		0	+	medium
6	Plp	Plp	+	+	+	+	4	s, 8, 10, 11	0	+	high
7	Plp	X	+	+	−	−	2	r, 13, 19, 21	0	n/a	medium
8	Plp	Pvx	+	+	+	−	3	r, 6, 10, 11	0	+	high
9	Plp	O3	+	+	−	−	2	r, 14, 31, 33	0	+	high
10	Pvx	Plp	+	+	+	−	3	r, 6, 8, 11	3	+	high
11	Pvx	Pvx	+	+	+	+	4	s, 6, 8, 10, 15, 17	3	+	high
12	Pvx	Met	+	+	−	−	2	r, 3, 4	3	+	high
13	Pvx	X	+	+	−	−	2	r, 7, 19, 20, 21	3	+	high
14	Pvx	O3	+	+	−	−	2	r, 9, 23, 31, 32, 33	3	+	high
**15**	Pvx	V	−	−	+	−	1	11, 17	3	−	medium
16	Pvx	Rdrp	+	−	−	−	1	r, 26, 27, 28	3	+	high
**17**	V	V	−	+	+	−	2	s, 15, 11	3	−	medium
18	X	Met	+	+	+	+	4	r, 1	0	n/a	high
19	X	Plp	+	−	+	−	2	r, 7, 13, 21	0	−	medium
20	X	X	+	+	+	+	4	s, 13, 21	0	+	high
21	X	Pvx	+	+	+	−	3	r, 7, 13, 19, 20	0	+	high
22	X	Hel	+	−	+	−	2	r, 24	0	+	high
23	X	O3	+	+	+	+	4	r, 14, 32, 33	0	+	high
24	Hel	X	+	+	−	−	2	r, 22	0	+	high
25	Hel	O3	+	−	−	−	1		0	+	medium
26	Rdrp	Plp	+	−	−	−	1	16, 27, 28	10	−	medium
27	Rdrp	X	+	+	−	−	2	16, 26, 28	10	−	medium
28	Rdrp	Pvx	+	+	−	−	2	r, 16, 26, 27	10	+	high
29	Rdrp	O3	+	+	−	−	2		10	+	high
**30**	Rdrp	Rdrp	−	+	+	−	2	s	10	−	medium
31	O3	Plp	−	−	+	−	1	r, 9, 14, 33	3	+	high
32	O3	X	+	+	+	+	4	r, 14, 23, 33	3	n/a	high
33	O3	Pvx	+	−	+	+	3	r, 9, 14, 23, 31, 33	3	+	high
34	O3	O3	+	+	+	+	4	s	3	+	high
35	O2	X	+	−	−	−	1		0	+	medium

NN, NC, CN, CC indicates the nature of fusion proteins by which an interaction was or not detected (+ or −, respectively). Redundant PPI in Y2H: Interactions that could be potentially reproduced by a) more than one vector pairing, b) reciprocal interaction (r, found in both bait-prey and prey-bait orientation) or c) overlapping constructs (indicated by their consecutive number). Possible only if the constructs P, V, X or Pvx are involved. (s): Self-interaction. The quality of interactions was classified from “high quality” to “basic quality”. “high quality”: Redundant interaction found in Y2H and LuMPIS. “medium quality”: Redundant interaction found in Y2H or LuMPIS. “basic quality”: No redundant interaction detected. Interactions No. 15, 17 and 30 were detected by combinations of C-terminal tagged proteins.

**Table 3:** Inter domain interactions of the HEV capsid protein.

InteractionNo.	Bait	Prey	NN	NC	CN	CC	No. Vectorcombinations	Comment
1	O2_FLd	O2_FLd	−	+	+	−	2	s, LuMPIS
2	O2_FLd	O2_P1P2	−	−	+	+	2	r
3	O2_FLd	O2_P2	−	−	+	−	1	r
4	O2_FLd	O2_S	−	+	+	+	3	
5	O2_FLd	O2_SP1	−	+	+	+	3	
6	O2_P1	O2_FLd	−	+	−	−	1	
7	O2_P1	O2_P1	−	+	+	−	2	s
8	O2_P1	O2_P1P2	−	+	+	−	2	
9	O2_P1	O2_S	−	−	+	+	2	
10	O2_P1	O2_SP1	−	+	−	−	1	
11	O2_P1P2	O2_FLd	−	+	−	−	1	r
12	O2_P1P2	O2_P1	−	+	+	−	2	
13	O2_P1P2	O2_P1P2	−	+	+	+	3	s
14	O2_P1P2	O2_P2	−	−	+	−	1	
15	O2_P1P2	O2_S	−	−	+	+	2	
16	O2_P2	O2_FLd	−	+	+	+	3	r
17	O2_P2	O2_P1P2	−	+	+	+	3	
18	O2_P2	O2_P2	+	+	+	+	4	r
19	O2_P2	O2_S	−	−	+	+	2	
20	O2_SP1	O2_P1	−	+	+	−	2	
21	O2_SP1	O2_P1P2	−	−	+	−	1	
22	O2_SP1	O2_S	−	+	+	−	2	
23	O2_SP1	O2_SP1	−	+	−	−	1	s

NN, NC, CN, CC indicate the nature of fusion proteins by which an interaction was or was not detected (+ or −, respectively). Redundant PPI in Y2H: Interactions that could be potentially reproduced by a) more than one vector pairing, b) reciprocal interaction (r, found in both bait-prey and prey-bait orientation) or c) overlapping constructs (indicated by their consecutive number). Possible only if the constructs P, V, X or Pvx are involved. (s): Self-interaction. Inter-domain interactions are multiply verified by overlapping constructs and were not retested by LuMPIS (except Interaction No. 1). Interactions were scored on 1 mM 3-AT.

**Table 4:** Network parameters in Hepatitis E, SARS and herpesviruses.

Network Parameters	HEVY2H	HEVLuMPIS	SARS	HSV-1	VZV	mCMV	EBV	KSHV
#Proteins	16	9	31	48	57	111	61	50
#Interactions	35	20	65	111	173	406	218	123
#Interactions (w/o self-interactions)	26	15	59	100	160	393	208	115
Average degree^a^	4.38	4.44	4.19	4.63	6.07	7.32	7.15	4.92
Average degree (w/o self-interactions)	3.25	3.33	3.81	4.17	5.61	7.08	6.82	4.60
Characteristic path length^b^	1.65	1.64	2.43	2.79	2.34	2.84	2.44	2.84
Diameter^c^	3.00	3.00	5.00	6.00	5.00	7.00	5.00	7.00
Clustering coefficient^d^	0.71	0.77	0.41	0.25	0.39	0.24	0.40	0.15
Enrichment over ER	2.44	1.39	2.94	2.53	3.65	3.68	3.35	1.45
Enrichment over ES	1.36	NA	0.99	0.79	1.00	1.23	1.14	0.69

^a^Average degree: number of interactions per proteins.

^b^Characteristic path length average shortest path length.

^c^Diameter: maximum shortest path length.

^d^Clustering coefficient and enrichment of clustering coefficient compared to the average of 1000 random graphs of the same size (ER) and 1000 random networks with the same degree distribution (ES). For the Hepatitis E LuMPIS network, enrichment over ES could not be calculated due to its small size. Self-interactions were not included for the computation of average clustering coefficients, characteristic path length and network diameter as well as enrichment values.
