# Supplementary material for: The Hepatitis E virus intraviral interactome
Source: Sci Rep. 2015 Oct 14;5:13872. doi: 10.1038/srep13872 (PMC4604457; doi:10.1038/srep13872)
Supplement: Supplementary Information [file srep13872-s1.pdf]

The Hepatitis E virus intraviral interactome

*Andreas Osterman[1,§]; Thorsten Stellberger[2,3,§]; Anna Gebhardt[1,†]; Marisa Kurz[1]; Caroline C. Friedel[4]; Peter Uetz[2,5]; Hans Nitschko[1]; Armin Baiker[3]; Maria G. Vizoso-Pinto[1,6]**

Supplementary Table 1. Overview of vectors used for drawing the HEV Interactome

Construct ID	Position (aa) in the respective ORFp	Description	Gateway entry clone	Y2H Expression vectors (N- and C-terminal fusions)	LUMPIS expression vectors
Met	56-237	Methyl-transferase	pENTR207-Met	pGBKT7g-Met<br>pGADT7g-Met<br>pGBKCg-Met<br>pGADCg-Met	pCR3.1NMBP<br>pCR3.1NeGFPluc
Y	216-432	Y domain	pENTR207-Y	pGBKT7g-Y<br>pGADT7g-Y<br>pGBKCg-Y<br>pGADCg-Y	pCR3.1NMBP<br>pCR3.1NeGFPluc
Plp	433-592	Papain-like cysteine protease	pENTR207-Plp	pGBKT7g-Plp<br>pGADT7g-Plp<br>pGBKCg-Plp<br>pGADCg-Plp	pCR3.1NMBP<br>pCR3.1NeGFPluc
V	712-778	Hyper-variable region	pENTR207-V	pGBKT7g-V<br>pGADT7g-V<br>pGBKCg-V<br>pGADCg-V	pCR3.1NMBP<br>pCR3.1NeGFPluc
X	785-942	X domain	pENTR207-X	pGBKT7g-X<br>pGADT7g-X<br>pGBKCg-X<br>pGADCg-X	pCR3.1NMBP<br>pCR3.1NeGFPluc
Pvx	433-942	Putative functional protease complex	pENTR207-Pvx	pGBKT7g-Pvx<br>pGADT7g-Pvx<br>pGBKCg-Pvx<br>pGADCg-Pvx	pCR3.1NMBP<br>pCR3.1NeGFPluc
Hel	960-1204	Helicase domain	pENTR207-Hel	pGBKT7g-Hel<br>pGADT7g-Hel<br>pGBKCg-Hel<br>pGADCg-Hel	pCR3.1NMBP<br>pCR3.1NeGFPluc

RdRp	1207-1695	RNA dependent RNA polymerase	pENTR207-RdRp	pGBKT7g-RdRp<br>pGADT7g-RdRp<br>pGBKCg-RdRp<br>pGADCg-RdRp	pCR3.1NMBP<br>pCR3.1NeGFPluc
O3	1-123	ORF 3	pENTR207-O3	pGBKT7g-O3<br>pGADT7g-O3<br>pGBKCg-O3<br>pGADCg-O3	pCR3.1NMBP<br>pCR3.1NeGFPluc
O2	1-660	ORF 2	pENTR207-O2	pGBKT7g-O2<br>pGADT7g-O2<br>pGBKCg-O2<br>pGADCg-O2	pCR3.1NMBP<br>pCR3.1NeGFPluc
O2_FLd	112-608	ORF2$^{112-608}$	pENTR207-O2_FLd	pGBKT7g-O2_FLd<br>pGADT7g-O2_FLd<br>pGBKCg-O2_FLd<br>pGADCg-O2_FLd	
O2_S	118-313	ORF2 S domain	pENTR207-O2_S	pGBKT7g-S<br>pGADT7g-S<br>pGBKCg-S<br>pGADCg-S	
O2_P1	314-453	ORF2 P1 domain	pENTR207-O2_P1	pGBKT7g-P1<br>pGADT7g-P1<br>pGBKCg-P1<br>pGADCg-P1	
O2_SP1	118-453	ORF2 S and P1 domain	pENTR207-O2_SP1	pGBKT7g-SP1<br>pGADT7g-SP1<br>pGBKCg-SP1<br>pGADCg-SP1	
O2_P2	454-661	ORF2 P2 domain	pENTR207-O2_P2	pGBKT7g-O2_P2<br>pGADT7g-O2_P2<br>pGBKCg-O2_P2<br>pGADCg-O2_P2	
O2_P1P2	314-661	ORF2 P1 and P2 domain	pENTR207-O2_P1P2	pGBKT7g-O2_P1P2<br>pGADT7g-O2_P1P2<br>pGBKCg-O2_P1P2<br>pGADCg-O2_P1P2	
